# Suppression of hyperinsulinaemia in growing female mice provides long-term protection against obesity

**DOI:** 10.1007/s00125-015-3676-7

**Published:** 2015-07-09

**Authors:** Nicole M. Templeman, Susanne M. Clee, James D. Johnson

**Affiliations:** Diabetes Research Group, Life Sciences Institute, Department of Cellular and Physiological Sciences, University of British Columbia, 2350 Health Sciences Mall, Vancouver, BC Canada V6T 1Z3

**Keywords:** Adolescence, Diet-induced obesity, Glucose homeostasis, Insulin, Knockout mice, Type 2 diabetes

## Abstract

**Aims/hypothesis:**

Hyperinsulinaemia is associated with obesity but its causal role in the onset of obesity remains controversial. In this study, we tested the hypothesis that transient attenuation of diet-induced insulin hypersecretion in young mice can provide sustained protection against obesity throughout adult life.

**Methods:**

Using ‘genetically humanised’ mice lacking both alleles of rodent-specific *Ins1*, we compared mice heterozygous for the ancestral insulin gene *Ins2* with *Ins2*^+/+^ controls. Female *Ins1*^−/−^:*Ins2*^+/−^ and *Ins1*^−/−^:*Ins2*^+/+^ littermates were fed chow or high-fat diet (HFD). Insulin secretion, metabolic health variables and body mass/composition were tracked for over 1 year. We examined islet function and adipose transcript levels of adipogenic, lipogenic and lipolytic genes at two time points.

**Results:**

In control *Ins1*^−/−^:*Ins2*^+/+^ mice, HFD resulted in elevated fasting and glucose-stimulated insulin secretion between 8 weeks and 27 weeks of age. Hyperinsulinaemia was reduced by nearly 50% in *Ins1*^−/−^:*Ins2*^+/−^ mice during this period, without lasting adverse effects on glucose homeostasis. This corresponded with attenuated weight gain and adiposity. White adipose tissue from *Ins1*^−/−^:*Ins2*^+/−^ mice had fewer large lipid droplets, although transcriptional changes were not detected. Importantly, *Ins1*^−/−^:*Ins2*^+/−^ mice remained lighter than *Ins1*^−/−^:*Ins2*^+/+^ littermates despite reaching an equivalent degree of hyperinsulinaemia on HFD by 52 weeks.

**Conclusions/interpretation:**

These data demonstrate that attenuation of hyperinsulinaemia in young, growing female mice provides a long-lasting protection against obesity. This protection persists despite a late-onset emergence of hyperinsulinaemia in HFD-fed *Ins1*^−/−^:*Ins2*^+/−^ mice. Given the evolutionary conserved roles of insulin, it is possible that suppressing hyperinsulinaemia early in life may have far-reaching consequences on obesity in full-grown adult humans.

**Electronic supplementary material:**

The online version of this article (doi:10.1007/s00125-015-3676-7) contains peer-reviewed but unedited supplementary material, which is available to authorised users.

## Introduction

Over 40 million children younger than 5 years of age were overweight worldwide in 2011 [1], and being overweight or obese during childhood and adolescence is a predictor of adult obesity [2, 3]. Obesity is a risk factor for type 2 diabetes, coronary heart disease, hypertension, atherosclerosis and some cancers; many of these diseases are traditionally associated with adulthood but are occurring with increasing prevalence in youth [4]. Hyperinsulinaemia and insulin resistance are two characteristics of the obese state that have been proposed to contribute to its detrimental effects on health [5, 6]. Circulating insulin levels are intimately related to systemic insulin responsiveness, and the most widely held paradigm posits that obesity leads to insulin resistance, causing a compensatory rise in insulin to prevent hyperglycaemia [5, 6]. However, clinical and experimental evidence suggests that hyperinsulinaemia can precede and promote obesity [7–11]. Drugs that suppress insulin secretion in hyperinsulinaemic obese rodents or humans lead to weight loss [12–14]. Obese individuals with the highest insulin levels respond best to diets that reduce postprandial glycaemia and insulinaemia whereas those with less-elevated insulin show equivalent weight loss on low-fat diets [15, 16]. Insulin is known to suppress lipolysis and stimulate lipogenesis in white adipose tissue (WAT) [17], and mouse models with reduced adipose tissue insulin signalling are protected against obesity [18, 19].

We exploited the existence of two rodent insulin genes (*Ins1* and *Ins2*) to genetically manipulate endogenous insulin production. Recent work in our laboratory demonstrated that continuous suppression of fasting hyperinsulinaemia through reducing *Ins1* dosage (in an *Ins2* null background) prevented diet-induced obesity in male mice [11]. However, *Ins1* is a rodent-specific gene, and there are differences in promoter elements and expression patterns between *Ins1* and *Ins2* [11, 20–23]. As it is unclear whether *Ins1* and *Ins2* have distinct roles, we felt it important to examine the effects of reduced *Ins2* dosage in the development of high-fat diet (HFD)-induced obesity.

Certain life stages are important for adipocyte hyperplasia and hypertrophy but the mechanisms controlling WAT expansion, and their timing, remain to be fully elucidated [24]. White adipocyte cell number is thought to stabilise towards the end of adolescence in non-obese humans and rodents [24], which suggests that conditions during this programming period could influence future adiposity. Indeed, adolescence has been identified as a key life stage for the development of obesity in humans, since the presence or onset of obesity during adolescence is associated with increased incidence of its associated morbidities in adults [25]. In our previous study, the *Ins1*^+/−^:*Ins2*^−/−^ genetic manipulation resulted in lifelong prevention of hyperinsulinaemia [11], which precluded an assessment of whether anti-obesity effects would persist without sustained repression of insulin. In the present study, we found that high-fat-fed female *Ins1*^−/−^:*Ins2*^+/−^ mice had reduced insulin secretion at a young age but by 1 year they had reached a degree of hyperinsulinaemia equivalent to that of high-fat-fed *Ins1*^−/−^:*Ins2*^+/+^ littermates. This provided a unique model with which to test the hypothesis that the reduction of insulin secretion in young, growing mice can provide long-term protection against diet-induced obesity.

## Methods

### Experimental animals

**Fig. 1:**
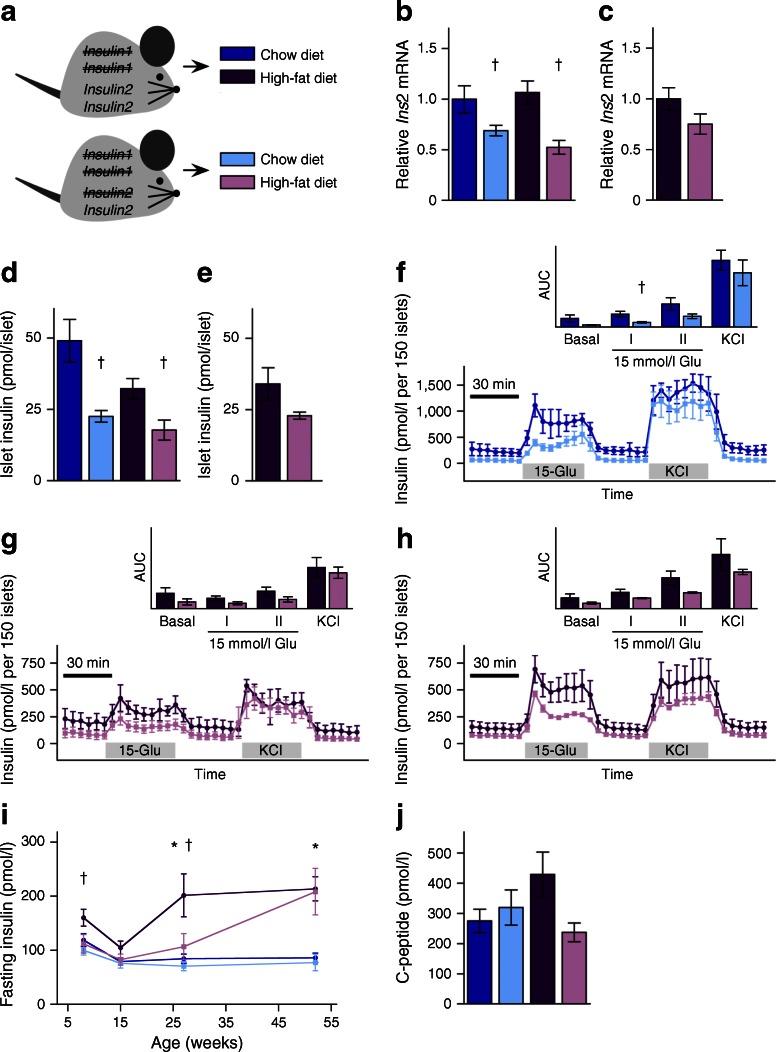
Transiently attenuated HFD-induced hyperinsulinaemia in *Ins1* ^−/−^:*Ins2* ^+/−^ mice. (**a**) Experimental design of *Ins1* ^−/−^:*Ins2* ^+/+^ and *Ins1* ^−/−^:*Ins2* ^+/−^ littermates fed CD or HFD. (**b**, **c**) Islet *Ins2* mRNA is corrected against β-actin mRNA and normalised to CD-fed *Ins1* ^−/−^:*Ins2* ^+/+^ mice at 25 weeks (*n* = 3–5) (**b**), or HFD-fed *Ins1* ^−/−^:*Ins2* ^+/+^ mice at 50 weeks (*n* = 3) (**c**). (**d**, **e**) Insulin content in islets from mice at 25 (**d**) and 50 weeks (**e**). (**f**–**h**) At 25 (**f**, **g**) and 50 weeks (**h**), insulin secretion by 150 islets perifused with basal 3 mmol/l glucose followed by stimulatory 15 mmol/l glucose (Glu) or 30 mmol/l KCl, with AUC (insets; *y*-axis units, pmol/l × min) depicted, including phases I/II of glucose stimulation (*n* = 3). (**i**, **j**) Fasted insulin (*n* = 17–21) (**i**) and C-peptide (*n* = 5–6) (**j**) at 27 weeks is from in vivo sampling. Dark blue, CD-fed *Ins1* ^−/−^:*Ins2* ^+/+^ mice; dark purple, HFD-fed *Ins1* ^−/−^:*Ins2* ^+/+^ mice; light blue, CD-fed *Ins1* ^−/−^:*Ins2* ^+/−^ mice; light purple, HFD-fed *Ins1* ^−/−^:*Ins2* ^+/−^ mice. Data are means ± SEM. *p ≤ 0.05, CD vs HFD; ^†^ *p* ≤ 0.05, *Ins1* ^−/−^:*Ins2* ^+/+^ vs *Ins1* ^−/−^:*Ins2* ^+/−^

All animal procedures were approved by the University of British Columbia Animal Care Committee, following Canadian Council for Animal Care guidelines. The *Ins1*-null and *Ins2*-null alleles were generated by Jami and colleagues [26]. Mice had mixed genetic background (predominately C57BL/6 and 129 strains). *Ins1*^−/−^:*Ins2*^+/+^ and *Ins1*^−/−^:*Ins2*^+/−^ female mice from each litter were weaned (at 3–4 weeks) and distributed (with matching body weights) between two diet assignments: a moderate-energy chow diet (CD) (25% fat content; LabDiet 5LJ5; PMI Nutrition International, St. Louis, MO, USA) or a high-energy HFD (58% fat content; Research Diets D12330; Research Diets, New Brunswick, NJ, USA) [11] provided ad libitum, except during fasting periods (Fig. 1a). Mice were housed under specific pathogen-free conditions at 21°C, on a 12 h light–dark cycle. We considered in our studies whether the dam or sire of individual mice had the disrupted *Ins2* allele, to control for possible parental imprinting [27, 28], but as no obvious parental effects were observed, we combined ‘parental groups’. Experimenters were blind to mouse genotype, diet (where possible), and parental group while performing and analysing each experiment.

### Glucose homeostasis and plasma analytes

Mice were fasted for 4 h during the light period to ensure a postprandial state for blood sampling. Fasting and glucose-stimulated (2 g/kg) insulin secretion was assessed, as well as blood glucose response to intraperitoneal injection of glucose (2 g/kg) or insulin analogue (0.75 U/kg of Humalog; Eli Lilly, Indianapolis, IN, USA). Plasma insulin was measured with a mouse insulin ELISA (Alpco Diagnostics, Salem, NH, USA) and C-peptide was measured in a subset of 27-week-old mice with a mouse C-peptide ELISA (Alpco Diagnostics). In plasma from 40-week-old mice, we measured total cholesterol (Cholesterol E kit; Wako Chemicals, Richmond, VA, USA), triacylglycerols (Serum Triacylglycerol kit; Sigma-Aldrich, St Louis, MO, USA) and NEFA levels (NEFA-HR(2) kit; Wako Chemicals), as well as leptin, resistin, interleukin 6, glucose-dependent insulinotropic polypeptide (GIP), peptide YY and glucagon, using a mouse magnetic bead panel assay utilising Luminex technology (Millipore, St Charles, MO, USA).

### Body composition and whole body metabolism

A subset of pups was weighed prior to weaning. After weaning, all mice were weighed weekly. Body composition was assessed using dual-energy x-ray absorptiometry (DEXA) (Lunar PIXImus densitometer; GE Medical Systems LUNAR, Madison, WI, USA). A subset of 17-week-old mice on HFD was evaluated in PhenoMaster metabolic cages (TSE Systems, Chesterfield, MO, USA), as described elsewhere [29]. Before data collection, mice were individually housed for at least 1 week and were allowed to become acclimated to the PhenoMaster cage environment for at least 4 days. Data were averaged from 48–84 h of continual acquisition.

### Murine INSULIN2 treatment

A cohort of mice were put on HFD at 15 weeks and subcutaneously implanted at 17 weeks with mini osmotic pumps (Alzet 2004; DURECT, Cupertino, CA, USA) designed to release murine INSULIN2 peptide (0.1 U/day; generously provided by Novo Nordisk, Bagsvaerd, Denmark) or vehicle for 28 days.

### Tissue analyses

Tissues were dissected from mice 4 h after fasting. Mice were from the 50-week-old HFD-fed mini osmotic pump group and 25-week-old CD or HFD groups. Islets were isolated as described [30], with minor modifications, and cultured at 37°C and 5% CO_2_ for at least 16 h in RPMI-1640 medium (Invitrogen, Burlington, ON, Canada) supplemented with 11 mmol/l glucose, 100 U/ml penicillin, 100 μg/ml streptomycin and FBS (10% vol./vol.). Islet perifusions were performed as described [31] using 150 size-matched islets per group. Insulin content was measured by ELISA from ten islets per mouse that were lysed at −20°C using 0.02 mol/l HCl in 70% ethanol and sonicated for 30 s.

Other tissues were weighed and flash-frozen in liquid nitrogen before being stored at −80°C, except for half of the gonadal WAT depot, which was fixed in 4% paraformaldehyde, embedded in paraffin and cut into 5 μm-thick sections that were stained for perilipin (antibody 9349S; Cell Signaling Technology, Danvers, MA, USA) using an Alexa fluor 488-conjugated secondary antibody (Life Technologies, Burlington, ON, Canada) and DAPI. Images with identical exposure times were taken with a Zeiss 200 M inverted microscope (Carl Zeiss, Oberkochen, Germany) and lipid droplet areas were determined using CellProfiler 2.1.0 (http://www.cellprofiler.org/) [32].

For real-time PCR, total RNA was extracted from islets using the Qiagen RNeasy Mini kit (Qiagen, Mississauga, ON, Canada), and from adipose tissue using acid guanidinium thiocyanate–phenol–chloroform extraction with TRIzol (Invitrogen) followed by Qiagen RNeasy Mini columns [33]. cDNA was generated using a qScript cDNA synthesis kit (Quanta Biosciences, Gaithersburg, MD, USA). Reactions were performed on default parameters of a StepOnePlus real-time PCR system (Applied Biosystems, Foster City, CA, USA), using TaqMan Fast Universal PCR or Fast SYBR Green Master Mixes (Applied Biosystems). See electronic supplementary material [ESM] Table 1 for primer details. Values were normalised using the ${2}^{-\Delta {\mathrm{C}}_{\mathrm{t}}}$ method.

### Statistical analyses

Statistical analyses were performed with SPSS 15.0 software and a critical α-level of *p* ≤ 0.05. Two-way ANOVA models were used to assess factors of genotype and diet and a significant interaction led to one-way ANOVAs comparing HFD-fed *Ins1*^−/−^:*Ins2*^+/+^ mice, CD-fed *Ins1*^−/−^:*Ins2*^+/+^ mice, HFD-fed *Ins1*^−/−^:*Ins2*^+/−^ mice and CD-fed *Ins1*^−/−^:*Ins2*^+/−^ mice, with Bonferroni corrections applied. ANCOVA was used to test energy expenditure with covariates of lean and fat mass. One-way ANOVAs or two-tailed *t* tests were used to analyse tissue data from the 50-week time point.

## Results

### Attenuated hyperinsulinaemia in young HFD-fed ***Ins1***^−/−^:***Ins2***^+/−^ mice

To characterise this novel model, we first examined the effect of reduced *Ins2* gene dosage on insulin mRNA, protein and secretion. Islets from 25-week-old *Ins1*^−/−^:*Ins2*^+/−^ mice predictably expressed significantly less *Ins2* than islets from the *Ins1*^−/−^:*Ins2*^+/+^ mice, although this difference was less marked in islets from HFD-fed mice at 50 weeks (Fig. 1b, c). No differences were observed in *Pdx1*, *Hnf1a*, *Slc2a2* or *Gck* expression at 50 weeks (data not shown). Insulin protein content was also reduced by ∼50% in *Ins1*^−/−^:*Ins2*^+/−^ islets compared with *Ins1*^−/−^:*Ins2*^+/+^ islets, at 25 weeks (Fig. 1d, e). Insulin secretion was modestly, but consistently, lower in islets isolated from *Ins1*^−/−^:*Ins2*^+/−^ mice, with a significant reduction in first-phase response to stimulatory glucose in chow-fed mice (Fig. 1f–h). Collectively, these data demonstrate that reduced *Ins2* dosage resulted in lower insulin production, although there is potential for age-dependent compensation with HFD-feeding.

**Fig. 2:**
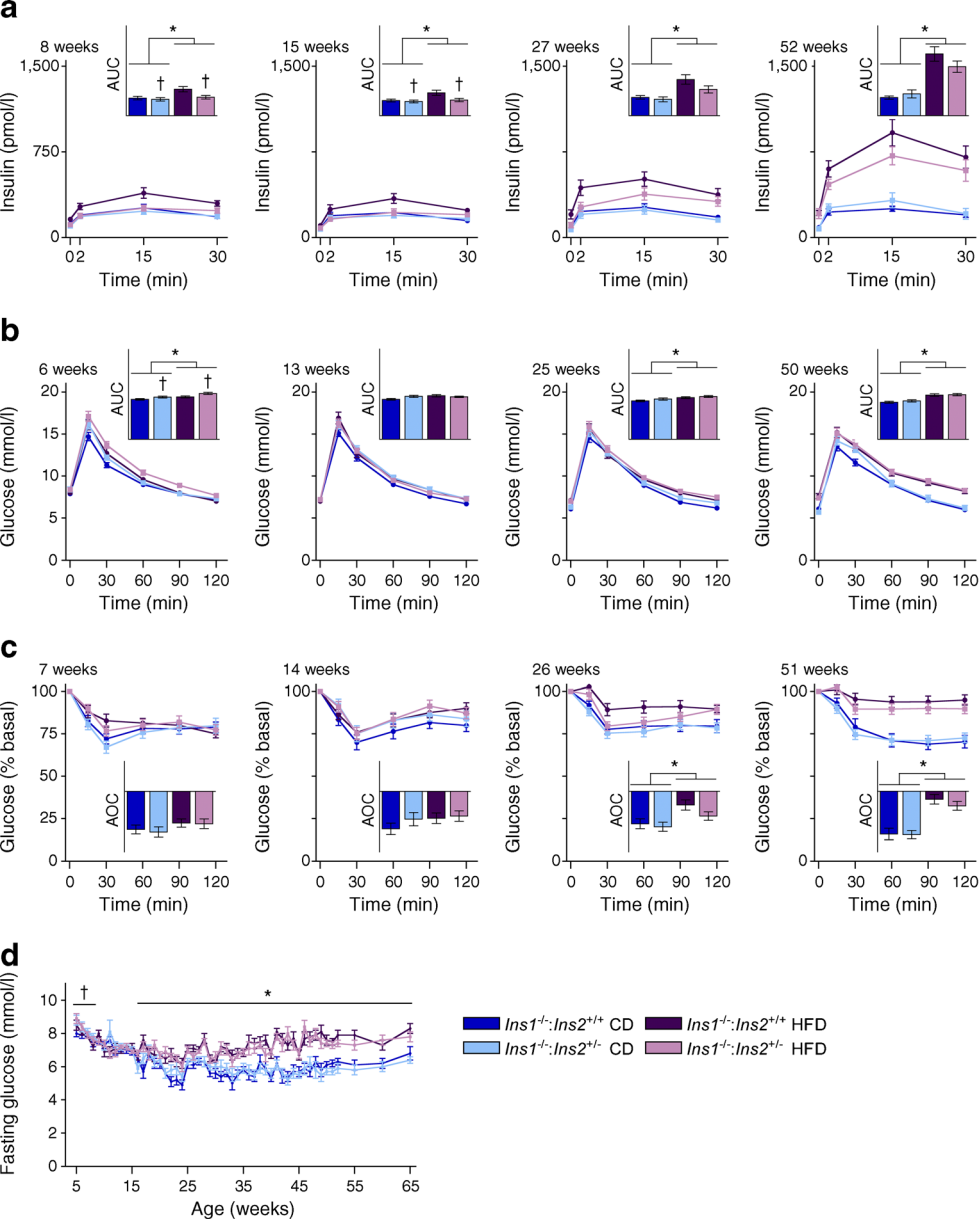
Longitudinal glucose homeostasis. Periodic measurements of glucose-stimulated insulin secretion (*n* = 17–21) (**a**), blood glucose responses to intraperitoneal glucose (*n* = 29–34) (**b**) and insulin analogue (*n* = 29–34) (**c**) are shown, together with AUC or area over curve (AOC) (insets, *y*-axis units, pmol/l × min [**a**], mmol/l × min [**b**] and % × min [**c**]) and fasted blood glucose (*n* = 15–18, most time points) (**d**). Dark blue, CD-fed *Ins1* ^−/−^:*Ins2* ^+/+^ mice; dark purple, HFD-fed *Ins1* ^−/−^:*Ins2* ^+/+^ mice; light blue, CD-fed *Ins1* ^−/−^:*Ins2* ^+/−^ mice; light purple, HFD-fed *Ins1* ^−/−^:*Ins2* ^+/−^ mice. Data are means ± SEM. *p ≤ 0.05, CD vs HFD; ^†^ *p* ≤ 0.05, *Ins1* ^−/−^:*Ins2* ^+/+^ vs *Ins1* ^−/−^:*Ins2* ^+/−^

Reduced fasting insulin was detected in *Ins1*^−/−^:*Ins2*^+/−^ mice at 8 weeks, compared with *Ins1*^−/−^:*Ins2*^+/+^ mice (Fig. 1i). Statistically significant HFD-induced fasting hyperinsulinaemia was observed in *Ins1*^−/−^:*Ins2*^+/+^ mice by 27 weeks and insulin levels remained elevated at 1 year (Fig. 1i). Importantly, *Ins1*^−/−^:*Ins2*^+/−^ mice were protected against HFD-induced fasting hyperinsulinaemia until at least 27 weeks (Fig. 1i). *Ins1*^−/−^:*Ins2*^+/−^ mice were unable to increase fasting C-peptide in response to HFD at this age, confirming that reduced basal insulin secretion, rather than increased insulin clearance, accounted for these differences (Fig. 1j). At all measured time points, HFD led to higher glucose-stimulated insulin secretion than CD, but at 8 and 15 weeks, glucose-stimulated insulin secretion was lower in *Ins1*^−/−^:*Ins2*^+/−^ mice than in *Ins1*^−/−^:*Ins2*^+/+^ littermates (Fig. 2a). However, in this mouse model, suppression of insulin secretion was not sustained since by 1 year of age *Ins1*^−/−^:*Ins2*^+/−^ female mice had equivalently high fasting and glucose-stimulated insulin levels when compared with the *Ins1*^−/−^:*Ins2*^+/+^ mice on HFD (Figs 1i, 2a).

### Longitudinal analysis of glucose homeostasis

We found that reduced *Ins2* dosage only had minor effects on glucose homeostasis (Fig. 2b–d). At 6 weeks, *Ins1*^−/−^:*Ins2*^+/−^ mice had slightly impaired glucose tolerance compared with *Ins1*^−/−^:*Ins2*^+/+^ littermates (Fig. 2b). As marginally elevated fasting glucose levels were observed in 5- to 8-week-old *Ins1*^−/−^:*Ins2*^+/−^ mice (Fig. 2d), insulin secretion may not be completely sufficient for normal glycaemic control in very young *Ins1*^−/−^:*Ins2*^+/−^ mice. However, these differences were only evident for a brief period (Fig. 2b, d), suggesting that there were no lasting negative repercussions in *Ins1*^−/−^:*Ins2*^+/−^ mice. There were only minor long-term HFD effects on glucose homeostasis (Fig. 2b). Moderate glucose intolerance in the HFD groups was associated with significant insulin resistance, with a notable decline at 26 weeks and beyond in HFD-fed vs CD-fed mice (Fig. 2c). By 16 weeks, all HFD-fed mice had elevated fasting blood glucose, compared with CD-fed mice (Fig. 2d). Therefore, any differences between HFD-fed *Ins1*^−/−^:*Ins2*^+/−^ and *Ins1*^−/−^:*Ins2*^+/+^ female mice would likely be driven by attenuated hyperinsulinaemia in young *Ins1*^−/−^:*Ins2*^+/−^ mice, rather than altered glucose homeostasis.

### Lasting protection against obesity in HFD-fed ***Ins1***^−/−^:***Ins2***^+/−^ mice

**Fig. 3:**
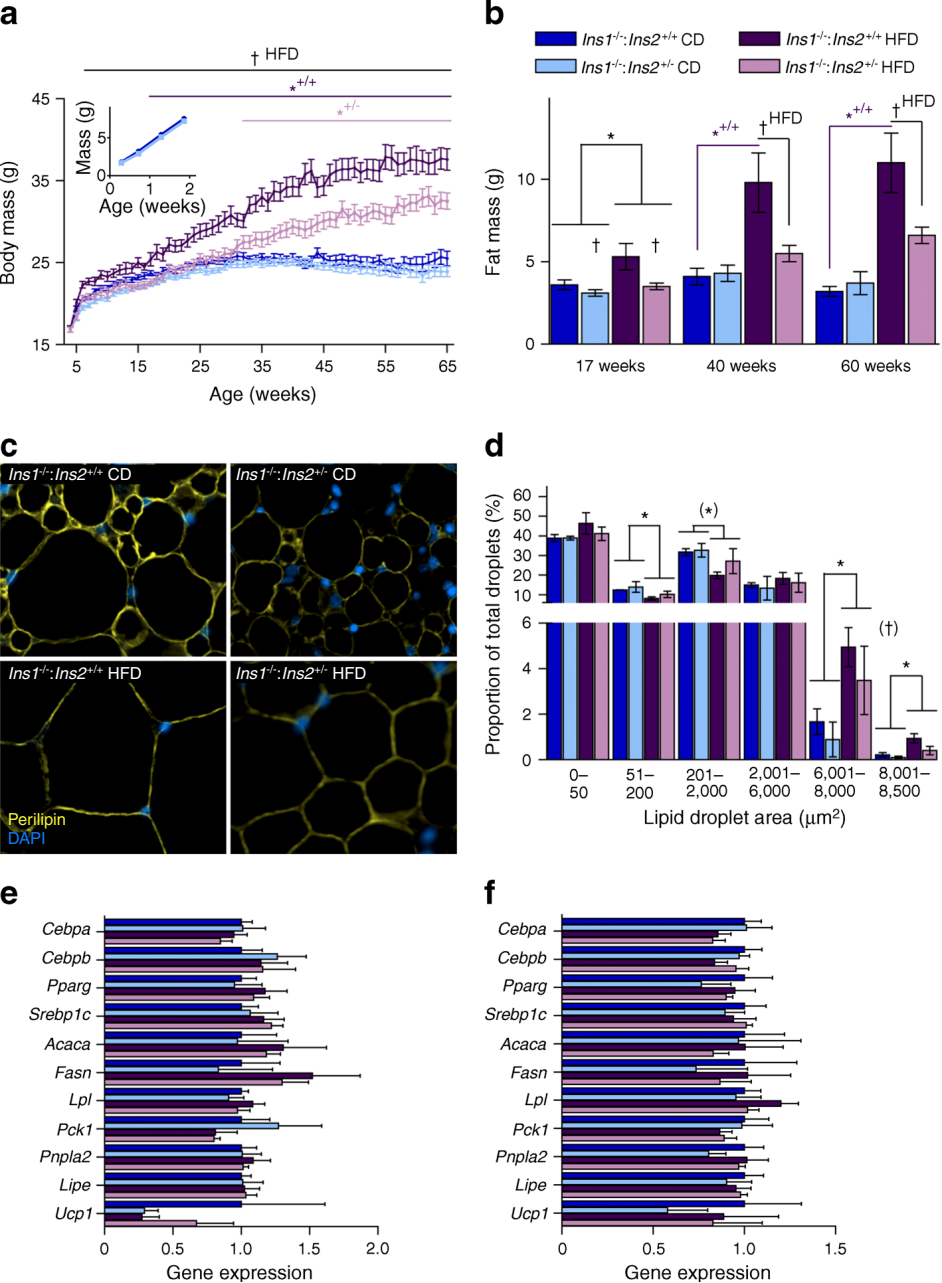
Attenuated obesity in HFD-fed *Ins1* ^−/−^:*Ins2* ^+/−^ mice. (**a**) Body mass was tracked in pups (*n* = 19–29, inset) and weaned mice (*n* = 29–33, most time points). (**b**) DEXA-measured fat mass is shown (*n* = 8–11). (**c**, **d**) Staining (magnification ×50) for perilipin (yellow) and DAPI (blue) in gonadal WAT of 25-week-old mice (**c**) is quantified (*n* = 3) in (**d**) as frequency per size category. (**e**, **f**) Gene expression in gonadal (**e**) and inguinal (**f**) WAT mRNA is corrected against *Hprt* and normalised to CD-fed *Ins1* ^−/−^:*Ins2* ^+/+^ mice. Dark blue, CD-fed *Ins1* ^−/−^:*Ins2* ^+/+^ mice; dark purple, HFD-fed *Ins1* ^−/−^:*Ins2* ^+/+^ mice; light blue, CD-fed *Ins1* ^−/−^:*Ins2* ^+/−^ mice; light purple, HFD-fed *Ins1* ^−/−^:*Ins2* ^+/−^ mice. Data are means ± SEM. *p ≤ 0.05, ^(^*^)^ *p* = 0.051, CD vs HFD; ^†^ *p* ≤ 0.05, ^(†)^ *p* = 0.054, *Ins1* ^−/−^:*Ins2* ^+/+^ vs *Ins1* ^−/−^:*Ins2* ^+/−^; *^+/+^ *p* ≤ 0.05, CD-fed vs HFD-fed *Ins1* ^−/−^:*Ins2* ^+/+^ mice; *^+/−^ *p* ≤ 0.05, CD-fed vs HFD-fed *Ins1* ^−/−^:*Ins2* ^+/−^ mice; ^†HFD^ *p* ≤ 0.05, HFD-fed *Ins1* ^−/−^:*Ins2* ^+/+^ vs *Ins1* ^−/−^:*Ins2* ^+/−^ mice

Next, we tested our primary hypothesis that reduced insulin levels early in life might attenuate diet-induced obesity. No significant mass differences were observed between *Ins1*^−/−^:*Ins2*^+/−^ and *Ins1*^−/−^:*Ins2*^+/+^ pups prior to weaning (Fig. 3a). However, weight differences between high fat-fed *Ins1*^−/−^:*Ins2*^+/−^ and *Ins1*^−/−^:*Ins2*^+/+^ littermates were apparent almost immediately after introduction of the HFD (Fig. 3a). While HFD-fed *Ins1*^−/−^:*Ins2*^+/+^ mice were significantly heavier than CD-fed *Ins1*^−/−^:*Ins2*^+/+^ littermates as early as 18 weeks, the HFD had no detectable effect on *Ins1*^−/−^:*Ins2*^+/−^ body mass until after 32 weeks (Fig. 3a). Furthermore, on the HFD, the *Ins1*^−/−^:*Ins2*^+/−^ mice were still significantly lighter than their *Ins1*^−/−^:*Ins2*^+/+^ littermates past 1 year of age (Fig. 3a), a time when fasting insulin levels were equivalent (Fig. 1i). At all time points studied, the HFD increased absolute fat mass (Fig. 3b) and proportional fat mass (data not shown) in *Ins1*^−/−^:*Ins2*^+/+^ mice. Moreover, HFD-fed *Ins1*^−/−^:*Ins2*^+/−^ mice showed a significant attenuation of adipose tissue expansion when compared with *Ins1*^−/−^:*Ins2*^+/+^ mice (Fig. 3b). These data demonstrate that lower circulating insulin in young *Ins1*^−/−^:*Ins2*^+/−^ mice corresponded with a delayed and reduced degree of HFD-induced weight gain and obesity.

To look for evidence of adipose programming underlying differences in obesity, we examined inguinal (subcutaneous) and gonadal (visceral) WAT depots in 25-week-old mice. In gonadal WAT, perilipin staining revealed that while all HFD-fed mice had relatively fewer small lipid droplets and more large lipid droplets than CD-fed mice, *Ins1*^−/−^:*Ins2*^+/+^ mice tended to have a greater proportion of the largest lipid droplets (Fig. 3c, d). However, there were no significant differences detected in transcript levels of measured WAT adipogenic, lipogenic or lipolytic genes at this age (Fig. 3e, f).

### Effects of HFD on circulating lipids and metabolic factors

**Fig. 4:**
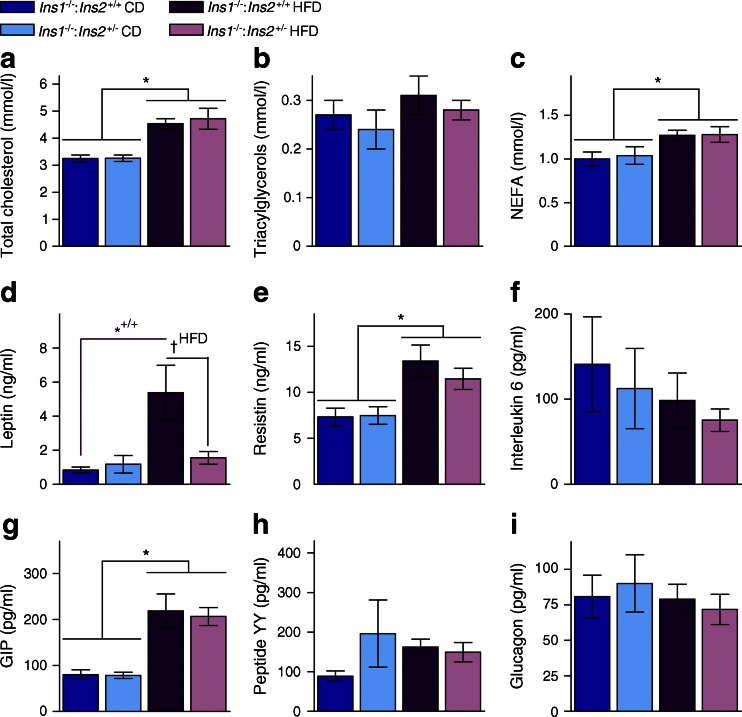
Plasma lipids and metabolic factors. Fasted levels of cholesterol (**a**), triacylglycerols (**b**), NEFA (**c**), leptin (**d**), resistin (**e**), interleukin 6 (**f**), GIP (**g**), peptide YY (**h**) and glucagon (**i**) in 40-week-old mice (*n* = 8–12). Dark blue, CD-fed *Ins1* ^−/−^:*Ins2* ^+/+^ mice; dark purple, HFD-fed *Ins1* ^−/−^:*Ins2* ^+/+^ mice; light blue, CD-fed *Ins1* ^−/−^:*Ins2* ^+/−^ mice; light purple, HFD-fed *Ins1* ^−/−^:*Ins2* ^+/−^ mice. Data are means ± SEM. *p ≤ 0.05, CD vs HFD; *^+/+^ *p* ≤ 0.05, CD-fed vs HFD-fed *Ins1* ^−/−^:*Ins2* ^+/+^ mice; ^†HFD^ *p* ≤ 0.05, HFD-fed *Ins1* ^−/−^:*Ins2* ^+/+^ vs *Ins1* ^−/−^:*Ins2* ^+/−^ mice

To further characterise the effects of lower insulin levels and attenuated obesity in HFD-fed *Ins1*^−/−^:*Ins2*^+/−^ mice, we measured lipids and metabolic factors in plasma from fasted 40-week-old mice. All HFD-fed mice had higher cholesterol and NEFA than CD-fed mice, but triacylglycerols were not significantly different (Fig. 4a–c). As expected based on body composition, only *Ins1*^−/−^:*Ins2*^+/+^ mice showed elevated leptin on HFD when compared with their chow-fed controls (Fig. 4d). However, both groups of HFD-fed mice had increased resistin levels (Fig. 4e). We did not detect significant differences in interleukin 6 (Fig. 4f). GIP levels were similarly elevated in all HFD-fed mice (Fig. 4g). No significant differences were detected in concentrations of peptide YY (Fig. 4h) or glucagon (Fig. 4i). Therefore, while adult *Ins1*^−/−^:*Ins2*^+/−^ mice had attenuated HFD-induced weight gain and adiposity (Fig. 3), they showed many similarities to HFD-fed *Ins1*^−/−^:*Ins2*^+/+^ mice with respect to circulating metabolic factors, except leptin. These observations support the concept that attenuated obesity in *Ins1*^−/−^:*Ins2*^+/−^ mice was due to the transient reduction of insulin, rather than other factors.

### Energy homeostasis in young mice

**Fig. 5:**
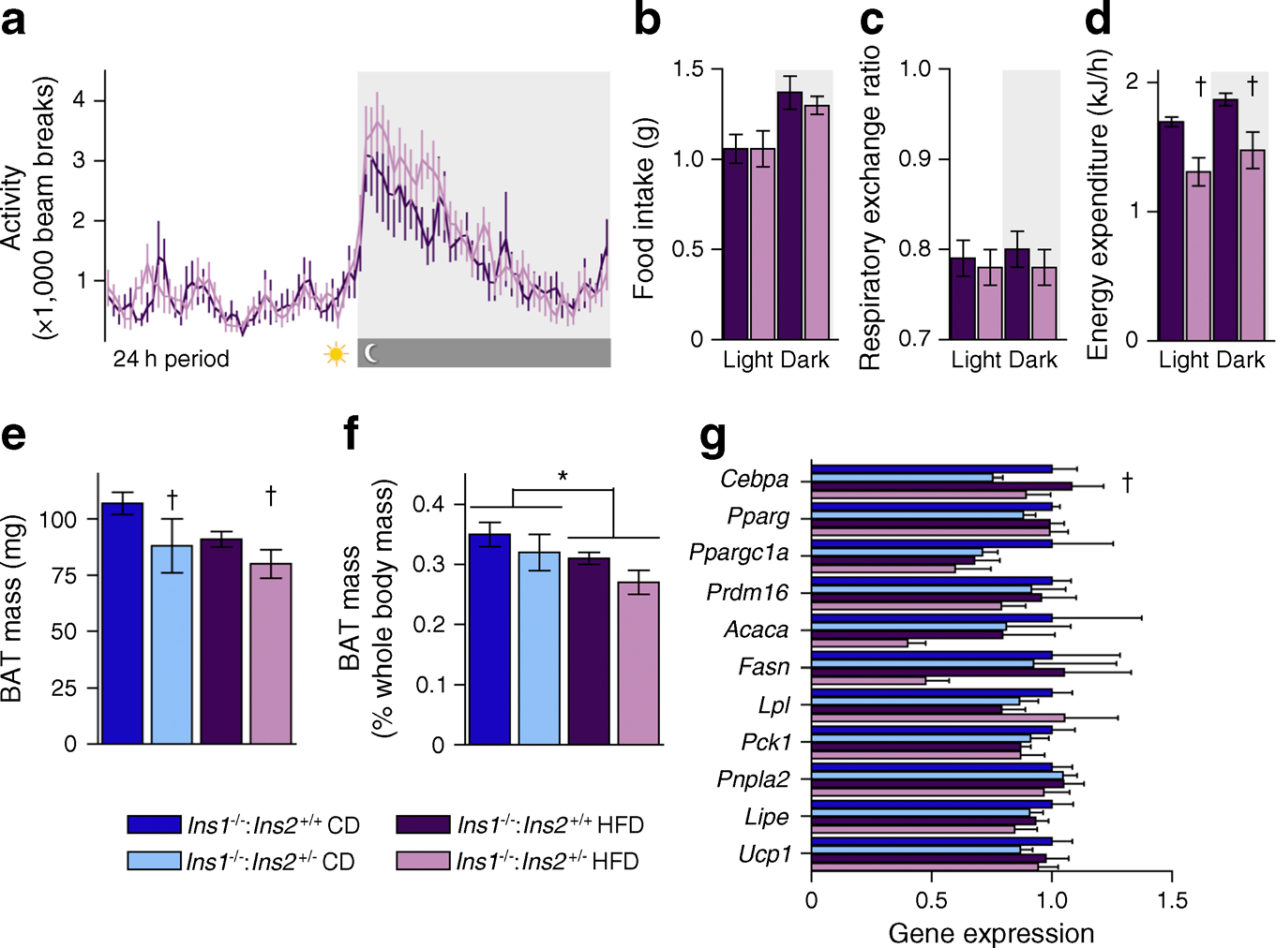
Energy homeostasis and brown adipose tissue. (**a**–**d**) In HFD-fed 17-week-old mice (*n* = 6–8), 24 h activity (**a**), food intake (**b**), respiratory exchange ratio (**c**) and energy expenditure (**d**) were averaged across 48–84 h; the dark period is shown in grey. (**e**, **f**) In 25-week-old mice (*n* = 5–7), BAT depot mass is shown as absolute values (**e**) and proportional to body mass (**f**). (**g**) mRNA levels of genes expressed in BAT are corrected against *Tbp* and normalised to levels in CD-fed *Ins1* ^−/−^:*Ins2* ^+/+^ mice. Dark blue, CD-fed *Ins1* ^−/−^:*Ins2* ^+/+^ mice; dark purple, HFD-fed *Ins1* ^−/−^:*Ins2* ^+/+^ mice; light blue, CD-fed *Ins1* ^−/−^:*Ins2* ^+/−^ mice; light purple, HFD-fed *Ins1* ^−/−^:*Ins2* ^+/−^ mice. Energy expenditure is shown as estimated marginal means ± SEM, adjusted for covariates of lean and fat mass; other data are simple means ± SEM. *p ≤ 0.05, CD vs HFD; ^†^ *p* ≤ 0.05, *Ins1* ^−/−^:*Ins2* ^+/+^ vs *Ins1* ^−/−^:*Ins2* ^+/−^

We examined the energy balance of HFD-fed *Ins1*^−/−^:*Ins2*^+/−^ and *Ins1*^−/−^:*Ins2*^+/+^ littermates at 17 weeks, during their growth divergence. Trends suggested that *Ins1*^−/−^:*Ins2*^+/−^ mice were more active than *Ins1*^−/−^:*Ins2*^+/+^ mice in the early hours of the dark cycle (Fig. 5a). While there were no significant differences in food intake or respiratory exchange ratios (Fig. 5b, c), *Ins1*^−/−^:*Ins2*^+/−^ mice exhibited reduced energy expenditure when compared with *Ins1*^−/−^:*Ins2*^+/+^ littermates (Fig. 5d). *Ins1*^−/−^:*Ins2*^+/−^ mice also had significantly reduced BAT mass compared with *Ins1*^−/−^:*Ins2*^+/+^ littermates at 25 weeks (*p* < 0.05, Fig. 5e; *p* = 0.102 when normalised to body mass, Fig. 5f), and lower BAT *Cebpa* expression (Fig. 5g). Overall, attenuated adiposity in HFD-fed *Ins1*^−/−^:*Ins2*^+/−^ mice was achieved despite decreased energy expenditure and BAT mass.

### Long-term effects of short-term INSULIN2 treatment

**Fig. 6:**
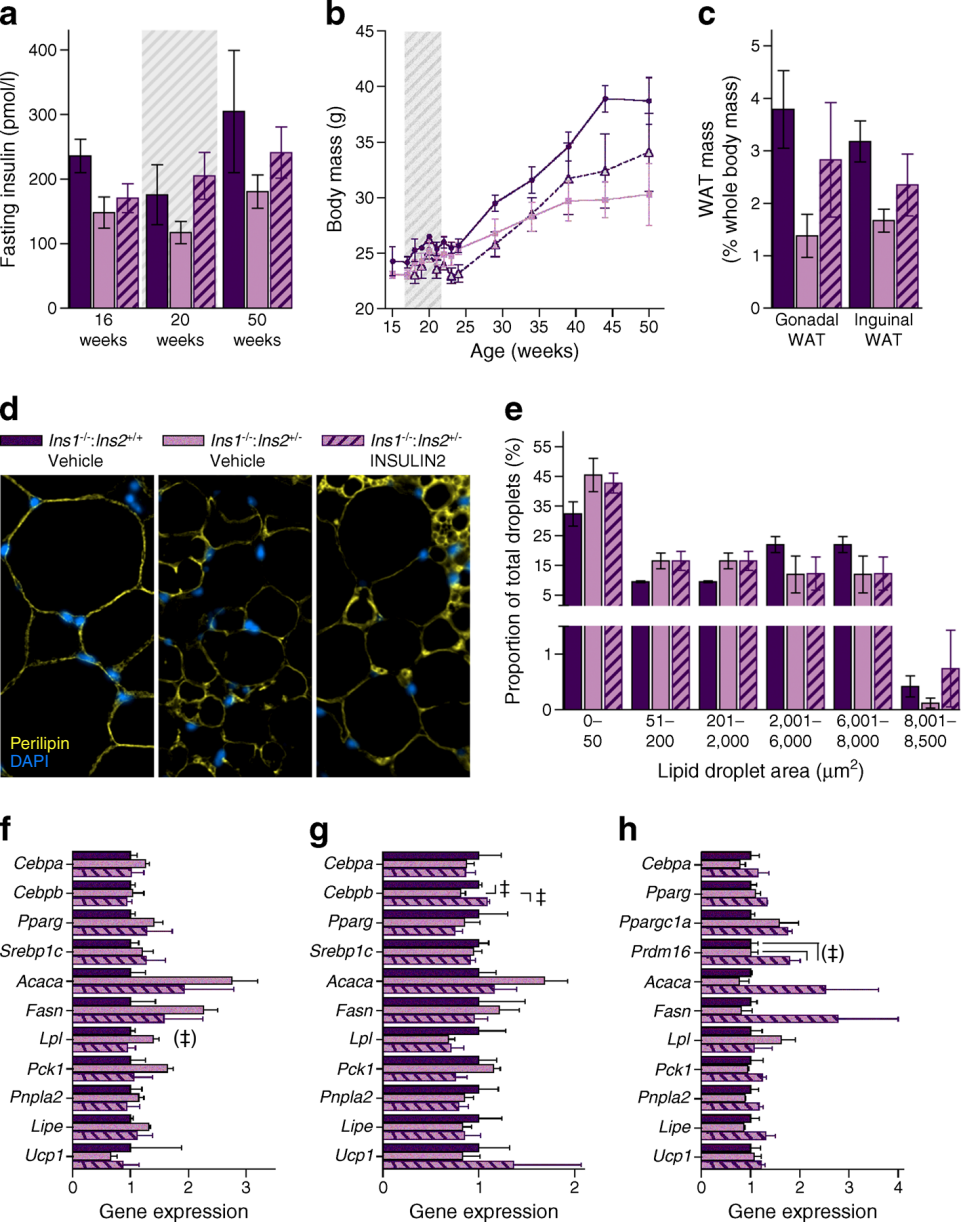
Long-term effects of HFD and INSULIN2. Mice were implanted with pumps releasing INSULIN2 or vehicle for 4 weeks (indicated by grey hatching). (**a**) Fasted insulin levels, *n* = 3. (**b**) Body mass, *n* = 3. (**c**–**e**) At 50 weeks, characterisation included WAT depot weight proportional to body mass (**c**) and gonadal WAT staining (magnification ×50) for perilipin (yellow) and DAPI (blue) (**d**) (*n* = 3; quantification is shown in **e** as frequency per size category). (**f**–**h**) mRNA levels of genes expressed in gonadal WAT (**f**), inguinal WAT (**g**) and BAT (**h**) are corrected against *Tbp* or *Hprt*, and normalised to levels in *Ins1* ^−/−^:*Ins2* ^+/+^ mice. Dark purple, vehicle-treated *Ins1* ^−/−^:*Ins2* ^+/+^ mice; light purple, vehicle-treated *Ins1* ^−/−^:*Ins2* ^+/−^ mice; hatched bars and triangles, INSULIN2-treated *Ins1* ^−/−^:*Ins2* ^+/−^ mice. Data are means ± SEM. ^‡^ *p* ≤ 0.05, for the indicated comparisons; ^(‡)^ *p* = 0.058 in (**f**) for group effect; and ^(‡)^ *p* ≤ 0.057 in (**h**) for the indicated comparisons

In a small experiment designed to independently assess the effect of elevated insulin levels during growth, HFD-fed 17-week-old mice were implanted with mini osmotic pumps secreting murine INSULIN2 or vehicle (control) for 4 weeks. INSULIN2-treated *Ins1*^−/−^:*Ins2*^+/−^ mice tended to reach fasting insulin levels similar to those of *Ins1*^−/−^:*Ins2*^+/+^ mice during the treatment, without immediate effects on body mass (Fig. 6a, b). However, by 1 year of age, *Ins1*^−/−^:*Ins2*^+/−^ mice that had been previously INSULIN2-treated tended to have body and WAT masses that were intermediate between vehicle-treated *Ins1*^−/−^:*Ins2*^+/−^ and *Ins1*^−/−^:*Ins2*^+/+^ mice (Fig. 6b, c). Regardless of INSULIN2 treatment status, all HFD-fed *Ins1*^−/−^:*Ins2*^+/−^ mice tended to exhibit relatively smaller lipid droplets than *Ins1*^−/−^:*Ins2*^+/+^ mice in gonadal WAT, with modest trends to suggest that INSULIN2 treatment may be associated with more of the largest droplets (Fig. 6d, e). Compared with the other groups, vehicle-treated *Ins1*^−/−^:*Ins2*^+/−^ mice tended to have higher transcript levels of genes associated with fatty acid uptake and lipogenesis in gonadal WAT (e.g. *p* = 0.058 for *Lpl*; Fig. 6f) and had lower inguinal WAT *Cebpb* mRNA than other groups (Fig. 6g). INSULIN2 treatment tended to be associated with increased BAT *Prdm16* expression (Fig. 6h). While this experiment did not definitively show that short-term INSULIN2 treatment was sufficient to promote obesity in *Ins1*^−/−^:*Ins2*^+/−^ mice, it points to the potential for lasting effects, and should catalyse future studies.

## Discussion

Our objective was to test the hypothesis that reducing insulin secretion by partial disruption of the *Ins2* gene would prevent diet-induced obesity. A transient attenuation of insulin hypersecretion in young, growing *Ins1*^−/−^:*Ins2*^+/−^ mice allowed us to test the secondary hypothesis that these anti-obesity effects would persist despite late-onset HFD-induced elevations in insulin. Our data indicate that, under these experimental conditions, reduced dosage of the ancestral *Ins2* gene can provide protection against obesity similar to that gained by reducing the dosage of rodent-specific *Ins1* [11]. Importantly, the current study also identified the growth period of adolescence and young adulthood as a potentially critical time to suppress insulin escalation.

It is an intriguing concept that there could be key interventional periods for influencing obesity and associated health risks [34]. In humans, the rate of BMI increase during pubescence and the maximum BMI during young adulthood (22–24 years) can both be stronger predictors of adiposity at mature adulthood (35–45 years) than adult lifestyle variables [35]. It has previously been shown that early-life manipulations, such as short-term insulin treatment in neonatal rats, leads to increased weight gain as well as impaired glucose tolerance and insulin responsiveness into adulthood [36]. Our experiments with short-term INSULIN2 treatment in *Ins1*^−/−^:*Ins2*^+/−^ mice also pointed to possible long-term effects on weight gain, although the limited period of insulin treatment could have led to impaired insulin sensitivity and a lasting elevation of insulin secretion, as previously seen [36]. Collectively, our investigation indicates that repression of hyperinsulinaemia in young, HFD-fed mice can attenuate obesity throughout adulthood.

Tissue analyses showed subtle dissimilarities in lipid droplet size distributions between *Ins1*^−/−^:*Ins2*^+/−^ and *Ins1*^−/−^:*Ins2*^+/+^ mice in the gonadal WAT at 25 weeks, implying possible divergence in adipocyte hypertrophy or hyperplasia, although no significant transcriptional changes were detected. By the age of 50 weeks, HFD-fed *Ins1*^−/−^:*Ins2*^+/−^ mice showed a tendency for increased expression of genes associated with fatty acid uptake and lipogenesis in gonadal WAT. This could indicate continued maintenance of adipocyte function and energy storage capacity, suggested to be metabolically beneficial in both humans and mice [37–40], and it may have been counteracted by exogenous INSULIN2 treatment. Interestingly, we did not detect elevated adipose *Ucp1* expression in *Ins1*^−/−^:*Ins2*^+/−^ female mice, unlike *Ins1*^+/−^:*Ins2*^−/−^ HFD-fed male mice [11]. Rather, interscapular depots of BAT were smaller in young *Ins1*^−/−^:*Ins2*^+/−^ female mice than in *Ins1*^−/−^:*Ins2*^+/+^ littermates, similar to the effect of knocking the insulin receptor out of BAT [41], and this may have contributed to the lower energy expenditure. Another caveat complicating the energy expenditure interpretation is that there were differences in body composition [42], and human studies have also shown that obese individuals may not show reduced total energy expenditure even if they are less active than lean individuals [43, 44]. Therefore, adipose-level changes distinct from those outlined in our previous study [11] may have contributed to attenuated adiposity in the HFD-fed *Ins1*^−/−^:*Ins2*^+/−^ female mice.

The HFD-fed *Ins1*^−/−^:*Ins2*^+/−^ female mice maintained protection against obesity into adulthood, despite the fact that their suppression of fasting insulin had reverted by 1 year. At 50 weeks of age, the islet *Ins2* mRNA and insulin levels of HFD-fed *Ins1*^−/−^:*Ins2*^+/−^ mice approached those of *Ins1*^−/−^:*Ins2*^+/+^ littermates. Although we could not elucidate the chronology of the relationship between this late-onset hyperinsulinaemia and insulin resistance, it is clear from our results that reducing adipose tissue expansion and weight gain cannot always prevent the decline in glucose tolerance and insulin sensitivity that is associated with high-fat feeding.

In conclusion, results from this investigation support the body of literature that places hyperinsulinaemia mechanistically upstream of diet-induced obesity. The growth period of adolescence and young adulthood may be a critical time for shaping future adiposity and our study demonstrates that, in mice, repression of insulin hypersecretion during this life stage can provide long-term protection against obesity. Interestingly, pubescence in humans is associated with a transient period of reduced blood glucose responsiveness to insulin, and elevated insulin secretion [45, 46]. It could be worthwhile to explore whether limiting insulin hypersecretion during this phase could have lasting anti-obesity effects in humans.

## Electronic supplementary material

ESM Table 1 (PDF 82 kb)

## Abbreviations

BAT: Brown adipose tissue
CD: Chow diet
DEXA: Dual-energy x-ray absorptiometry
GIP: Glucose-dependent insulinotropic polypeptide
HFD: High-fat diet
WAT: White adipose tissue

## References

[1] World Health Organization (2013). Obesity and overweight, fact sheet 311.

[2] Whitaker RC, Wright JA, Pepe MS, Seidel KD, Dietz WH (1997). Predicting obesity in young adulthood from childhood and parental obesity. N Engl J Med.

[3] Magarey AM, Daniels LA, Boulton TJ, Cockington RA (2003). Predicting obesity in early adulthood from childhood and parental obesity. Int J Obes.

[4] Olshansky SJ, Passaro DJ, Hershow RC (2005). A potential decline in life expectancy in the United States in the 21st century. N Engl J Med.

[5] Kahn BB, Flier JS (2000). Obesity and insulin resistance. J Clin Invest.

[6] Prentki M, Nolan CJ (2006). Islet β cell failure in type 2 diabetes. J Clin Invest.

[7] Genuth SM, Przybylski RJ, Rosenberg DM (1971). Insulin resistance in genetically obese, hyperglycemic mice. Endocrinology.

[8] Odeleye OE, de Courten M, Pettitt DJ, Ravussin E (1997). Fasting hyperinsulinemia is a predictor of increased body weight gain and obesity in Pima Indian children. Diabetes.

[9] Sigal RJ, El-Hashimy M, Martin BC (1997). Acute postchallenge hyperinsulinemia predicts weight gain: a prospective study. Diabetes.

[10] Ishikawa M, Pruneda ML, Adams-Huet B, Raskin P (1998). Obesity-independent hyperinsulinemia in nondiabetic first-degree relatives of individuals with type 2 diabetes. Diabetes.

[11] Mehran AE, Templeman NM, Brigidi GS (2012). Hyperinsulinemia drives diet-induced obesity independently of brain insulin production. Cell Metab.

[12] Alemzadeh R, Jacobs W, Pitukcheewanont P (1996). Antiobesity effect of diazoxide in obese zucker rats. Metabolism.

[13] Alemzadeh R, Langley G, Upchurch L (1998). Beneficial effect of diazoxide in obese hyperinsulinemic adults. J Clin Endocrinol Metab.

[14] Lustig RH, Greenway F, Velasquez-Mieyer P (2005). A multicenter, randomized, double-blind, placebo-controlled, dose-finding trial of a long-acting formulation of octreotide in promoting weight loss in obese adults with insulin hypersecretion. Int J Obes.

[15] Pittas AG, Das SK, Hajduk CL (2005). A low-glycemic load diet facilitates greater weight loss in overweight adults with high insulin secretion but not in overweight adults with low insulin secretion in the CALERIE trial. Diabetes Care.

[16] Ebbeling CB, Leidig MM, Feldman HA (2007). Effects of a low-glycemic load vs low-fat diet in obese young adults: a randomized trial. JAMA.

[17] Saltiel AR, Kahn CR (2001). Insulin signalling and the regulation of glucose and lipid metabolism. Nature.

[18] Katic M, Kennedy AR, Leykin I (2007). Mitochondrial gene expression and increased oxidative metabolism: role in increased lifespan of fat-specific insulin receptor knock-out mice. Aging Cell.

[19] Boucher J, Mori MA, Lee KY (2012). Impaired thermogenesis and adipose tissue development in mice with fat-specific disruption of insulin and IGF-1 signalling. Nat Commun.

[20] Deltour L, Leduque P, Blume N (1993). Differential expression of the two nonallelic proinsulin genes in the developing mouse embryo. Proc Natl Acad Sci U S A.

[21] Deltour L, Vandamme J, Jouvenot Y (2004). Differential expression and imprinting status of *Ins1* and *Ins2* genes in extraembryonic tissues of laboratory mice. Gene Expr Patterns.

[22] Hay CW, Docherty K (2006). Comparative analysis of insulin gene promoters: implications for diabetes research. Diabetes.

[23] Meur G, Qian Q, da Silva Xavier G (2011). Nucleo-cytosolic shuttling of FoxO1 directly regulates mouse *Ins2* but not *Ins1* gene expression in pancreatic beta cells (MIN6). J Biol Chem.

[24] Berry R, Jeffery E, Rodeheffer MS (2014). Weighing in on adipocyte precursors. Cell Metab.

[25] Dietz WH (1994). Critical periods in childhood for the development of obesity. Am J Clin Nutr.

[26] Duvillié B, Cordonnier N, Deltour L (1997). Phenotypic alterations in insulin-deficient mutant mice. Proc Natl Acad Sci U S A.

[27] Deltour L, Montagutelli X, Guenet J-L, Jami J, Paldi A (1995). Tissue- and developmental stage-specific imprinting of the mouse proinsulin gene, *Ins2*. Dev Biol.

[28] Giddings SJ, King CD, Harman KW (1994). Allele specific inactivation of insulin 1 and 2, in the mouse yolk sac, indicates imprinting. Nat Genet.

[29] Lee KTY, Karunakaran S, Ho MM, Clee SM (2011). PWD/PhJ and WSB/EiJ mice are resistant to diet-induced obesity but have abnormal insulin secretion. Endocrinology.

[30] Salvalaggio PRO, Deng S, Ariyan CE (2002). Islet filtration: a simple and rapid new purification procedure that avoids ficoll and improves islet mass and function. Transplantation.

[31] Dror V, Nguyen V, Walia P, Kalynyak TB, Hill JA, Johnson JD (2007). Notch signalling suppresses apoptosis in adult human and mouse pancreatic islet cells. Diabetologia.

[32] Lamprecht MR, Sabatini DM, Carpenter AE (2007). CellProfiler: free, versatile software for automated biological image analysis. BioTechniques.

[33] Cirera S (2013). Highly efficient method for isolation of total RNA from adipose tissue. BMC Res Notes.

[34] Lawlor DA, Chaturvedi N (2006). Treatment and prevention of obesity – are there critical periods for intervention?. Int J Epidemiol.

[35] Guo SS, Huang C, Maynard LM (2000). Body mass index during childhood, adolescence and young adulthood in relation to adult overweight and adiposity: the Fels Longitudinal Study. Int J Obes Relat Metab Disord.

[36] Harder T, Rake A, Rohde W, Doerner G, Plagemann A (1999). Overweight and increased diabetes susceptibility in neonatally insulin-treated adult rats. Endocr Regul.

[37] Hoffstedt J, Förster D, Löfgren P (2007). Impaired subcutaneous adipocyte lipogenesis is associated with systemic insulin resistance and increased apolipoprotein B/AI ratio in men and women. J Intern Med.

[38] Roberts R, Hodson L, Dennis AL (2009). Markers of de novo lipogenesis in adipose tissue: associations with small adipocytes and insulin sensitivity in humans. Diabetologia.

[39] Phan J, Reue K (2005). Lipin, a lipodystrophy and obesity gene. Cell Metab.

[40] Kim J-Y, van de Wall E, Laplante M (2007). Obesity-associated improvements in metabolic profile through expansion of adipose tissue. J Clin Invest.

[41] Guerra C, Navarro P, Valverde AM (2001). Brown adipose tissue-specific insulin receptor knockout shows diabetic phenotype without insulin resistance. J Clin Invest.

[42] Butler AA, Kozak LP (2010). A recurring problem with the analysis of energy expenditure in genetic models expressing lean and obese phenotypes. Diabetes.

[43] Bandini LG, Schoeller DA, Dietz WH (1990). Energy expenditure in obese and nonobese adolescents. Pediatr Res.

[44] Ekelund U, Åman J, Yngve A, Renman C, Westerterp K, Sjöström M (2002). Physical activity but not energy expenditure is reduced in obese adolescents: a case–control study. Am J Clin Nutr.

[45] Goran MI, Gower BA (2001). Longitudinal study on pubertal insulin resistance. Diabetes.

[46] Hannon TS, Janosky J, Arslanian SA (2006). Longitudinal study of physiologic insulin resistance and metabolic changes of puberty. Pediatr Res.

