# Supplementary material for: Suppression of hyperinsulinaemia in growing female mice provides long-term protection against obesity
Source: Diabetologia. 2015 Jul 9;58(10):2392–402. doi: 10.1007/s00125-015-3676-7 (PMC4572061; doi:10.1007/s00125-015-3676-7)
Supplement: Supplementary file 1 — (PDF 82 kb) [file 125_2015_3676_MOESM1_ESM.pdf]

**ESM Table 1. Primer sequences used for real-time PCR mRNA quantification.**

| Gene | | Forward primer (5' to 3') | Reverse primer (5' to 3') |
|---|---|---|---|
| TaqMan Assays | | | |
| *Ins2* | Insulin 2 | GAAGTGGAGGACCCACAAGTG | GATCTACAATGCCACGCTTCTG |
| *Actb* | ß-actin[1] | | |
| SYBR Green Primer Sets | | | |
| *Pdx1* | Pancreatic and duodenal homeobox 1 | GACCTTTCCCGAATGGAACC | GTTCCGCTGTGTAAGCACC |
| *Hnf1a* | Hepatocyte nuclear factor 1-α | GCACACCCATGAAGACACAG | CCTGTGGGCTCTTCAATCAG |
| *Slc2a2* | GLUT-2 | CTGTGTCCAGCTTTGCAGTG | CCATCAAGAGGGCTCCAGTC |
| *Gck* | Glucokinase | GTTTTGTGTCGCAGGTGGAG | GTGGACACGCTTTCACAGG |
| *Actb* | ß-actin | TGCGTGACATCAAAGAGAAG | GATGCCACAGGATTCCATA |
| *Cebpa* | CCAAT/enhancer-binding protein-α | CCGGGAGAACTCTAACTC | GATGTAGGCGCTGATGT |
| *Cebpb* | CCAAT/enhancer-binding protein-ß | GCAAGAGCCGCGACAAG | GGCTCGGGCAGCTGCTT |
| *Pparg* | Peroxisome proliferator-activated receptor-γ | AGATTCTCCTGTTGACCCAGA | ACAGAGCTGATTCCGAAGTTG |
| *Pgc1a* | PPAR-γ coactivator 1-α | GTGCCATATCTTCCAGTGACC | GGTTGGCTTTATGAGGAGGA |
| *Prdm16* | PR domain containing 16 | CTCAAGTACATCCGTGTAGCG | CTTTCACATGCACCAACAGTTC |
| *Srebp1c* | Sterol regulatory element-binding protein 1-c | CGGAAGCTGTCGGGGTAG | GTTGTTGATGAGCTGGAGCA |
| *Acaca* | Acetyl-CoA carboxylase α | GCCTCTTCCTGACAAACGAG | TGACTGCCGAAACATCTCTG |
| *Fasn* | Fatty acid synthase | TGGGTTCTAGCCAGCAGAGT | ACCACCAGAGACCGTTATGC |
| *Lpl* | Lipoprotein lipase | GTGACCGATTTCATCAAGTTTGGAG | GACGGACACAAAGTTAGCACCAC |
| *Pck1* | Phosphoenolpyruvate carboxykinase-1 | GTATCATCTTTGGTGGCCGTA | TGATCTTGCCCTTGTGTTCTG |
| *Pnpla2* | Adipose triglyceride lipase | AACACCAGCATCCAGTTCAA | GGTTCAGTAGGCCATTCCTC |
| *Lipe* | Hormone-sensitive lipase | ACCGAGACAGGCCTCAGTGTG | GAATCGGCCACCGGTAAAGAG |
| *Ucp1* | Uncoupling protein 1 | CCTGGCAGATATCATCACCTTC | TCCCTAGGACACCTTTATACCT |
| *Hprt* | Hypoxanthine guanine phosphoribosyl transferase | TCCTCCTCAGACCGCTTTT | CCTGGTTCATCATCGCTAATC |
| *Tbp* | TATA box binding protein | TTCACCAATGACTCCTATGACC | CAAGTTTACAGCCAAGATTCACG |

[1]Commercial primer assay (Catalog number 4352341E, Applied Biosystems)
Other primers/PrimeTime qPCR assays are from Integrated DNA Technologies, Toronto, ON, Canada.
